# The global response: how cities and provinces around the globe tackled COVID-19 outbreaks in 2021 – authors’ reply

**DOI:** 10.1016/j.lansea.2023.100183

**Published:** 2023-03-21

**Authors:** Nityanand Jain, Zeeshan Ali Khan, Tungki Pratama Umar, William Jau, I-Chun Hung, Nguyen Thanh An, Ho-Wei Lin, Kai-Yang Chen, Nguyen Tien Huy

**Affiliations:** a Faculty of Medicine, Riga Stradinš University, 16 Dzirciema Street, Riga, LV-1007, Latvia; b Online Research Club (https://www.onlineresearchclub.org/), Nagasaki, Japan; c Shadan Institute of Medical Sciences, Hyderabad, India; d Faculty of Medicine, Sriwijaya University, Palembang, 30114, Indonesia; e School of Medicine, College of Medicine, Taipei Medical University, Taipei, Taiwan; f Faculty of Medicine, College of Medicine and Pharmacy, Duy Tan University, Da Nang, 550000, Viet Nam; g Institute for Research and Training in Medicine, Biology and Pharmacy, Duy Tan University, Da Nang, 550000, Viet Nam; h School of Tropical Medicine and Global Health, Nagasaki University, Japan

We would like to thank Ngo et al., for expressing their interest in our work.^1^ In our recent work, we looked at the preventive measures that were undertaken by various cities and provinces across the globe to prevent the spread of COVID-19 infection.^2^

In their correspondence, Ngo et al., have raised potential concerns in association with the data presented and certain definitions used in the paper. Upon receipt of their letter, we revisited our data sources. Herein, we provide a point-by-point response to the concerns raised by Ngo et al.^1^

First, Ngo et al., referred to the Statistical Yearbook of Vietnam 2021, which was published online on Aug 01, 2022 (https://www.gso.gov.vn/en/data-and-statistics/2022/08/statistical-yearbook-of-2021/). However, our publication was available online on June 22, 2022, which predates the source mentioned. The data source used for the population of Ho Chi Minh City (HCMC) was Worldometer (as mentioned in the footnotes of Table 1; https://www.worldometers.info/world-population/vietnam-population/). Worldometer shows a rounded-off population of 3.5 million for Ho Chi Minh City.

Nonetheless, upon verification with the Statistical Yearbook of Vietnam 2020, which was published on June 30, 2021, the correct rounded-off population should be 9.2 million (9.2276 million). Hence, the correct number of confirmed cases per 100,000 population in Table 2 should be 4675.93.

Regarding the data source for daily confirmed COVID-19 cases in Manila City, Philippines, the data source has been moved to the following new link (Department of Health, Republic of the Philippines) - https://drive.google.com/drive/folders/1e1MXKap1XXYTqbcMLodmDH2yx_aSy0dc; accessed Feb 03, 2023.

Similarly, for the population census for New Delhi, we would refer again to Worldometer (as mentioned in the footnotes of Table 1; https://www.worldometers.info/world-population/india-population/), which mentions the rounded-off population of Delhi as 10.9 million. We thank Ngo et al., for noting the error in nomenclature. The data represented in the paper corresponds with Delhi (Union Territory/NCT) and not the city of New Delhi.

Second, Ngo et al., raised concern about the calculation of number of hard lockdown days in Ho Chi Minh City. In Table 3, we mentioned that HCMC was under hard lockdown for 69 days following the Directive 16 of the Prime Minister, which came into effect from July 09, 2021. However, we considered the end point as Sept 15, 2021 based on local author's experiences and news reports supporting claims of partial redemption of non-essential services in certain sectors of the city (https://e.vnexpress.net/news/news/hcmc-begins-to-ease-covid-restrictions-4357337.html). As mentioned, although the Directive 16 was extended for two more weeks (till Sept 30, 2021; https://letranlaw.com/insights/ho-chi-minh-city-extends-lockdown-till-september-end-and-pilots-covid-green-cards/?fbclid=IwAR1aLWm9yaa2q3By9ksQIpmPQm3HN_-nN1wkRNHiFQcCPozV3rUSxJ1MEpg), postal and telecommunications services, computer stores, stationary stores, take-away catering services, supporting agricultural production, veterinary facilities, maintenance and repair of construction works, transportation, machinery and provision of components and parts serving such activities and services related to food production were allowed to be operated from 0600 h to 2100 h daily (https://moit.gov.vn/tin-tuc/dia-phuong/tp-hcm-tiep-tuc-gian-cach-den-30-9-mot-so-loai-hinh-kinh-doanh-duoc-hoat-dong-lai.html?fbclid=IwAR3qP1mn8bNJc26qe_IJ23GpstFP_CwocGTFMBhcqIVOF1IhCnfGNrV5PIM), which cannot be considered as a hard lockdown as mentioned in our article: an all-stay-at-home restriction with only essential businesses open. Hence, we would argue that the data provided in Table 3 is correct.

Furthermore, the advisory team constituted by HCMC differs from other taskforces established in other cities in the means that the HCMC advisory team could only advice and propose solutions to the relevant authorities but lacked administrative powers. Consider Bangkok's Metropolitan Authority (BMA), which had administrative powers to issue closure orders. Furthermore, the taskforces in other cities were directly chaired by the Ministers/Governors directly.

However, it should be noted that Ho Chi Minh City did set-up an administrative taskforce for COVID-19 on Nov 12, 2021 (https://vietreader.com/news/51758-ho-chi-minh-city-launches-task-force-on-covid-19-pandemic-control.html), which was outside the investigated study period. As mentioned in the methods section, the study period investigated in the present study ended on 31st October 2021.

The hotline 1022 referred to by Ngo et al., is a much more generic hotline, which has been used also for reporting breakdowns relating to public infrastructure (roads, water pipes, drainage, traffic lights, electricity poles), although it was released in the very early phases of the COVID-19 pandemic in April/May 2020 (https://1022.tphcm.gov.vn/gioi-thieu). During the pandemic, the service was available for offering support and providing guidance for HCMC COVID-19 patients isolating at home. The hotline was also used for consulting on COVID-19 prevention and other health-related issues.

As mentioned in the news “The toll-free call center (1022) was jointly established by the Department of Information and Communications and the Department of Health amid a rising number of infections and people in self-quarantine who needed to be supported by medical professionals, the helpline provides instructions on handling various situations that arise whilst monitoring the disease, taking care of severe cases, exercising, and using over-the-counter drugs.” (https://e.vnexpress.net/news/life/trend/hcmc-hotline-with-medical-professionals-helps-ease-strain-on-covid-frontlines-4335577.html).

Although the hotline could be used for reporting violations/issues related to lockdown measures, it was not the hotline's sole purpose. In fact, as recently as January 2023, the hotline was expanded also to receive complaints from international and domestic tourists regarding local tourism products and services (https://vietnamnet.vn/en/hcmc-extends-hotline-service-to-serve-tourists-2097143.html).

Conversely, consider Jakarta's Posko Hotline PPKM (PPKM Hotline Command Post) at 110/081113110110, which was specifically constituted in relation to the pandemic so the public can contact them using telephone or WhatsApp (https://corona.jakarta.go.id/id/posko-hotline-ppkm).

Third, regarding the inclusion of RT-PCR and RATs in the laboratory tests, as mentioned in the text (page 9 of manuscript), “All cities/provinces except Tokyo (Table 5), relied on using mass testing strategies comprising both rapid antigen tests and standard RT-PCRs.” The number of laboratory tests was gathered from official websites of relevant national reporting authorities. As mentioned in the footnotes of the table, the WHO “had previously suggested a positivity rate of around 3–12% as a general benchmark of adequate testing, along with recommending that test positivity should remain at 5% or lower for 14 days before regions reopen." The point raised by the Ngo et al., has already been addressed in the manuscript.

Fourth, we agree with the number of hospital beds in Ho Chi Minh City to be at 38,712. There has been a typographical error. Regarding number of doctors per 1000 inhabitants, such information was mostly available at national levels at not at city levels for other cities. Hence, to maintain homogeneity we used country-level statistics for that parameter.

Fifth, Ngo et al., pointed towards the Hayat-Vax vaccine's approval in Vietnam. We would like to draw attention to the fact that Hayat-Vax vaccine is the same vaccine as Sinopharm's Covilo vaccine, also known as BiBP inactivated vaccine (https://hayatbiotech.com/g42-announces-new-jv-with-sinopharm-cnbg-to-produce-hayat-vax-vaccine-in-the-uae/), the approval date for which is mentioned in Table 8 for Ho Chi Minh City. The vaccine is simply filled, packaged, and labelled in the United Arab Emirates (UAE) and not produced in the UAE. This is different from India's Covishield Vaccine, which is also manufactured in India (https://www.seruminstitute.com/product_covishield.php) and has been approved separately by the international authorities. Hence, Hayat-Vax was not mentioned separately in the table.

Next, in their letter, Ngo et al., asked to consider reporting clinical trials that are ongoing for COVID-19 vaccines. We would like to differ from the point raised. The aim of the study was to compare control measures and the testing strategies that were adopted in the cities/provinces. As we mentioned in the text “Since the issuance of emergency use authorization (EUA) did not necessarily correlate with the availability of vaccines, it would be difficult to estimate the role of more vs. fewer vaccines in the market.” Based on this, we believe that ongoing COVID-19 vaccine clinical trials offer no additional insight or information to provide a complete picture of vaccination record in the respective countries.

Finally, Ngo et al., expressed their disagreement to the definitions of “duration of outbreak” and “major outbreak” used in the paper. We agree regarding differences in population sizes of the cities but at the same time highlight the fact that the size of the city is not such a big factor in implementing control over COVID-19 spread.

Considering the example between Bangkok and Gauteng, Ngo et al., correctly note the population difference of three times between cities. However, as mentioned in Table 2, Bangkok despite being 3 times smaller, experienced larger number of outbreak days (155) compared to Gauteng (132) and the cumulative number of COVID-19 cases in Bangkok is just 1.5 times smaller than Gauteng. If size of the city was a reliable enough parameter, then the difference in cumulative numbers should have been also close to 2.5–3 times and not 1.5 times. Clearly, in comparison between these two cities/provinces, Gauteng was able to successfully implement control strategies. Like the point raised above, we do not think that our definition leads to a paradox since Manila City was effectively able to keep the COVID-19 cases under control as also noted by its way smaller number of cumulative COVID-19 cases.

We agree that our definition could have been expanded to include other parameters, something also acknowledged in the discussion section. However, we like to point out that there has been no previous study at the time of data collection that could be used as a benchmark or reference in defining a major COVID-19 outbreak. Our study provides insights into the fact that factors such as population density and total population size of a city are not necessarily the defining features for estimating the spread of COVID-19 infections in the city. If the suggested notion were to be applied, then logic would dictate that since the cumulative number of cases for Jakarta (677,805) is more than that for Gauteng (632,925), then population for Jakarta would also be higher. However, that is not the case. In fact, Jakarta is roughly 1.5 times smaller in population in comparison with Gauteng. Furthermore, Jakarta's population density is estimated at 15,900 people/km^2^ (https://www.bps.go.id/indicator/12/141/1/population-density-by-province.html) compared with Gauteng's 870 people/km^2^ (https://www.gcis.gov.za/sites/default/files/docs/gcis/1.%20Land%20and%20its%20people%20.pdf). This means that Jakarta, which has 18.27 times higher population density should also have much larger number of cases than Gauteng. However, this was not the case. This clearly shows that no two cities are identical (as also mentioned in discussion) in terms of implementation of control strategies and jurisdictional approaches.

Regarding inclusion of parameters like death rates, intensive care unit (ICU) bed utilisation etc., we would like to draw attention towards the definition of an outbreak. According to the WHO's definition, the suggested parameters are not included when defining an outbreak. WHO defines a disease outbreak as the “occurrence of cases of disease in excess of what would normally be expected in a defined community, geographical area or season” (https://www.emro.who.int/health-topics/disease-outbreaks/index.html). Since WHO definition depends only upon “cases of disease” we believe the definitions used in the study are sound and valid.

Based on the above-mentioned corrections and points of discussion, the authors would like to thank Ngo et al., for their time and meticulous observations. We have made the required corrections in the research article. We would also like the readers to note that the above discussion and corrections does not alter the main conclusions drawn in the present study.

## Contributors

NJ revisited the original source data and prepared the present document. ZKA, TPU, WJ, ICH, NTA, HWL, and KYC validated and provided suggestions for the discussion and the sources presented in the document. NTH supervised the team and was responsible for project administration and funding. All authors agreed to the contents of the manuscript and read the final version for publication.

## Declaration of interests

None.
